# Alterations in gonadotropin, apoptotic and metabolic pathways in granulosa cells warrant superior fertility of the Dummerstorf high fertility mouse line 1

**DOI:** 10.1186/s13048-023-01113-5

**Published:** 2023-02-04

**Authors:** Carolin Lisa Michaela Ludwig, Simon Bohleber, Rebecca Lapp, Alexander Rebl, Eva Katrin Wirth, Martina Langhammer, Ulrich Schweizer, Joachim M. Weitzel, Marten Michaelis

**Affiliations:** 1 Research Institute for Farm Animal Biology (FBN), Dummerstorf, Germany; 2 Institut für Biochemie und Molekularbiologie (IBMB), Rheinische Friedrich-Wilhelms-Universität Bonn, Bonn, Germany; 3 Department of Endocrinology and Metabolism, Charité - Universitätsmedizin Berlin, corporate member of Freie Universität Berlin, Humboldt-Universität zu Berlin, Berlin, Germany; 4 DZHK (German Centre for Cardiovascular Research), partner site Berlin, Berlin, Germany

**Keywords:** Follicular survival, FSH signaling, Granulosa cells, High-fertility, Ovulation rate

## Abstract

The development and maturation of ovarian follicles is a complex and highly regulated process, which is essential for successful ovulation. During recent decades, several mouse models provided insights into the regulation of folliculogenesis. In contrast to the commonly used transgenic or knockout mouse models, the Dummerstorf high-fertility mouse line 1 (FL1) is a worldwide unique selection experiment for increased female reproductive performance and extraordinary high fertility. Interactions of cycle-related alterations of parameters of the hypothalamic pituitary gonadal axis and molecular factors in the ovary lead to improved follicular development and therefore increased ovulation rates in FL1 mice. FL1 females almost doubled the number of ovulated oocytes compared to the unselected control mouse line. To gain insights into the cellular mechanisms leading to the high fertility phenotype we used granulosa cells isolated from antral follicles for mRNA sequencing. Based on the results of the transcriptome analysis we additionally measured hormones and growth factors associated with follicular development to complement the picture of how the signaling pathways are regulated. While IGF1 levels are decreased in FL1 mice in estrus, we found no differences in insulin, prolactin and oxytocin levels in FL1 mice compared to the control line. The results of the mRNA sequencing approach revealed that the actions of insulin, prolactin and oxytocin are restricted local to the granulosa cells, since hormonal receptor expression is differentially regulated in FL1 mice. Additionally, numerous genes, which are involved in important gonadotropin, apoptotic and metabolic signaling pathways in granulosa cells, are differentially regulated in granulosa cells of FL1 mice.

We showed that an overlap of different signaling pathways reflects the crosstalk between gonadotropin and growth factor signaling pathways, follicular atresia in FL1 mice is decreased due to improved granulosa cell survival and by improving the efficiency of intracellular signaling, glucose metabolism and signal transduction, FL1 mice have several advantages in reproductive performance and therefore increased the ovulation rate. Therefore, this worldwide unique high fertility model can provide new insights into different factors leading to improved follicular development and has the potential to improve our understanding of high fertility.

## Introduction

In polytocous species such as mice the number of ovulated oocytes is an important parameter for the reproductive performance. Before ovulation can occur, the oocytes have to undergo several highly regulated developmental sequences. This process, called folliculogenesis, is a complex process in which multiple organ systems are involved [1]. The foundation for subsequent follicular development is the non-growing pool of primordial follicles. After transition from the primordial follicle to the growing preantral follicle, the follicles achieve antral and finally preovulatory stage. In contrast to preantral follicles, which are characterized by gonadotropin-independent growth, antral follicles are highly differentiated endocrine structures under the control of the hormones of the hypothalamic pituitary gonadal axis (HPG axis), containing the oocyte surrounded by multiple layers of granulosa cells (GCs), the fluid filled antrum and thecal cells. Aside from gonadotropins, various other factors, including the insulin-like growth factor 1 (IGF1) and insulin, are involved in the follicular development [1–4]. However, not all follicles succeed in folliculogenesis. Less than 1% of the initially formed primordial follicles ovulate as mature follicles [5]. All others undergo an apoptotic process called atresia. Although atresia can occur at every stage of folliculogenesis, most follicles fail at the antral stage due to apoptosis of the GCs [6, 7]. Hence, the decision of whether a follicle is selected for ovulation or undergoes atresia is mainly made in GCs. The follicle stimulating hormone (FSH) is one of the most important key regulators in controlling antral follicle development and many transgenic and knockout mouse models demonstrate the great importance of FSH in antral follicle formation [1, 5, 8, 9]. Especially the role of FSH in promoting GC proliferation and inhibiting follicular atresia is remarkable [10–13]. FSH signaling is mediated by the G protein coupled FSH receptor (FSHR), leading to activation of signaling pathways such as protein kinase A (PKA), phosphatidylinositol-4,5-bisphosphate 3 kinase (PI3K)/protein kinase B (AKT) and extra cellular-regulated kinases (ERK1/2), which in turn regulate GC survival, apoptosis, growth and differentiation. Furthermore steroidogenesis is regulated by the FSH induced signaling pathways [1, 9]. FSH is indispensable for antral follicle development [14], and improving ovulation rates by administration of exogenous FSH is widely accepted and a common method in reproductive medicine as well as in research. However, while administration of FSH or substances that simulate FSH effects initially increase ovulation rates, several mouse models demonstrate that long-term overexpression might have negative effects on the fertility and health. Although activating mutations with increased *Fshr* activity in knock-in mice resulted in enhanced GC proliferation, these mice are infertile and are characterized by a loss of small follicles and the development of hemorrhagic cysts [15]. Mc Tavish et al. (2007) created transgenic mice overexpressing *Fsh*. Although these transgenic mice initially ovulated a higher number of oocytes, they suffered from accelerated reproduction failure and premature infertility [16]. In contrast to these transgenic mouse models the Dummerstorf high fertility mouse line 1 (FL1) represents a different approach to gain insights into the physiology of high fertility and the achievement of increased ovulation rates. FL1 mice are neither transgenic nor knockout mice, but are selected for increased litter size and total birth weight of the litters for more than 200 generations. During the selection process, FL1 mice almost doubled the number of pups per litter compared to the unselected control line (ctrl) with no signs of growth retardation in the offspring [17–19]. FL1 mice ovulate approximately twice as many oocytes compared to ctrl mice [20, 21]. Although the assumption that FL1 mice increased their ovulation rate due to higher FSH levels might be argumentative at first glance, the opposite is the case. Surprisingly, FSH levels in FL1 mice are decreased by 50% compared to ctrl mice [21]. Consequently, we have to reconsider the role of high FSH levels. Although it is undeniable that FSH is indispensable in antral follicle formation [14], this mouse model demonstrates that high ovulation rates can obviously not only be attributed to increased FSH levels.

Previous data of our laboratory indicate that the follicular development is improved in FL1 mice [21]. However, the cellular mechanisms leading to such increased ovulation rates despite decreased FSH levels remain mainly unclear. Thus, the aim of this study is to detect mechanisms and signaling pathways responsible for the achievement of the high fertility phenotype. Since FSH is known to be the major player in promoting GC proliferation in antral follicles, we isolated GCs from large antral follicles and performed a holistic gene expression approach. In addition we measured several endocrine parameters associated with follicular growth and GC differentiation to draw a global picture of how the signaling pathways are regulated and to provide insights into this alternative and highly efficient way of follicular development.

## Results

**Fig. 1:**
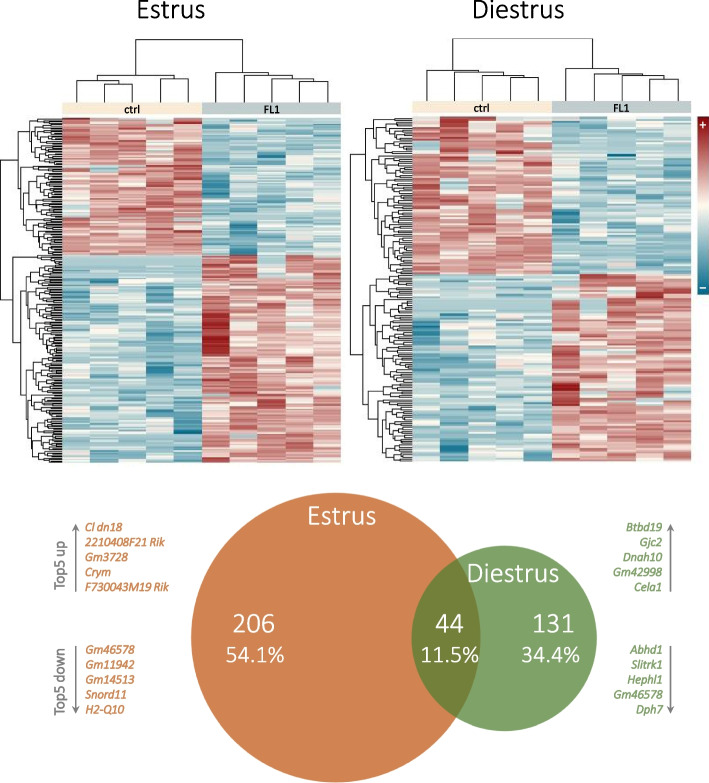
Hierarchical clustering dendrograms of differentially expressed genes (log2 FC) in (**A**) estrus and in (**B**) diestrus of FL1 and ctrl mice. High and low expression intensities are represented by red and blue color, respectively (see scale on the right margin). **C** A Venn diagram illustrates the absolute number and the percentage of DE features in the estrus and diestrus of FL1 mice relative to the expression values obtained for ctrl mice. The overlap depicts the proportion of DE genes shared by both mouse lines. The diagram was calculated based on the official gene symbols

In the present study we analyzed the gene expression in GCs to obtain an overview of the mechanisms that result in the improved follicular development of FL1 mice. The transcriptome analysis of GCs in estrus and diestrus from ctrl and FL1 mice revealed significant line-specific expression patterns (Fig. 1). In estrus 250 genes were differentially expressed (151 genes stronger expressed and 99 genes weaker expressed with q ≤ 0.05) in GCs of FL1 mice compared to ctrl mice. In diestrus 175 genes were differentially expressed (95 genes stronger expressed and 80 genes weaker expressed with q ≤ 0.05) in GCs of FL1 mice compared to ctrl mice.

A selection of differentially expressed genes, which are involved in different traits of female reproduction and folliculogenesis, is shown in Table 1.

**Table 1:** List of differentially expressed genes associated with reproductive traits in estrus and diestrus

Gene	ΔLog2FC	
	Estrus	Diestrus
G-protein coupled receptors		
*Oxtr*	3.2**	
*Lhcgr*	3.0*	
Transmembrane receptors		
*Prlr*	2.2*	
*Sfrp4*	1.3*	
Ligand-dependent nuclear receptors		
*Rora*	2.5*	
Enzymes		
*Kl*	6.8*	
*Vnn1*	2.1***	
*Acsl4*	1.5*	
*Hsd11b2*		1.3**
*Setd3*	1.2*	
*Hsd3b1*	0.9*	
*Cyp1b1*	−2.2***	
Growth factors		
*Agt*	−1.8***	
Kinases		
*Map 3 k14*	6.8***	
*Pik3c2g*	2.5**	
*Sgk1*	1.8**	
Phosphatases		
*Dusp23*	3.0***	2.6**
*Dusp2*		6.2*
*Inpp5d*		6.8*
Transporter		
*Star*	2.1*	2.3***
Cytokines		
*Timp1*	1.5**	

### Differentially regulated canonical pathways in FL1 mice versus ctrl mice

A canonical pathway analysis using the IPA Knowledge Base was performed to obtain insights in the molecular mechanisms underlying the increased reproductive performance of FL1 mice (Table 2 and Table 3).

**Table 2:** Differentially regulated functional pathways in GCs of FL1 compared to ctrl mice in estrus. The canonical pathway analysis is calculated based on the IPA Ingenuity Knowledge Base. Official gene symbols are sorted by the fold change, starting with the lowest Log2FC

Canonical pathway	*p*-value	z-score	Involved genes	
			Stronger expression	Weaker expression
Gluconeogenesis	1.73E-03	n.a.	*Gapdh, Me1*	*Me2*
Inhibition of matrix metalloproteases	5.58E-03	n.a.	*Timp1*	*Mmp11, Lrp1*
LXR/RXR activation	5.78E-03	−0.5	*Pltp, Apof, Lyz*	*Apoa4, Agt*
Xenobiotic metabolism general signaling pathway	1.07E-02	2.2	*Map3k14, Pik3c2g, Mgst2, Maf, Gsto1*	
Inositol phosphate metabolism	1.33E-02-3.53E-02	0.5–0.8	*Ptpn22, Ptpn22, Dusp23, Pik3c2g*	*Inpp5b, Sgpp2*
Insulin receptor signaling	4.13E-02	2.0	*Ppp1r14a, Pic3c2g, Sgk1*	*Inpp5b*
Myo-inositol biosynthesis	4.54E-02	n.a.		*Isyna1*

**Table 3:** Differentially regulated functional pathways in GCs of FL1 compared to ctrl mice in diestrus. The canonical pathway analysis is calculated based on the IPA Ingenuity Knowledge Base. Official gene symbols are sorted by the fold change, starting with the lowest Log2FC

Canonical pathway	*p*-value	z-score	Involved genes	
			Stronger expression	Weaker expression
RAR activation	1.03E-03	n.a.	*Smarcd3, Crabp2, Aldh1a1*	*Scand1, Rpl7a, Rarg*
Inositol phosphate metabolism	3.37E-03- 1.02E-02	1.0–1.3	*Inpp5d, Dusp2, Dusp23, Atp1a1*	*Ephx2*
Oxytocin signaling pathway	2.10E-02	1.3	*Ptgfr, Ppara, Pla2g12a, Pla2g4a*	*Cacna2d1*
Xenobiotic metabolism signaling	2.37E-02	n.a.	*Mgst2, Hs3st1, Maf, Aldh1a1*	*Scand1*
p38 MAPK signaling	2.99E-02	n.a.	*Pla2g12a, Pla2ga4*	*Mef2d*

Several pathways are differentially regulated in FL1 mice in estrus as well as in diestrus compared to ctrl mice. A number of genes associated with the inositol phosphate metabolism (*Ptpn22, Ppp1r14a, Dusp23, Inpp5b and Sgpp2* in estrus; *Inpp5d*, *Dusp2*, *Dusp23*, *Atp1a1* and *Ephx2* in diestrus) are differentially regulated in GCs of FL1 mice compared to ctrl mice.

The activation of the retinoic acid receptor (RAR) respectively the heterodimer retinoid X receptor (RXR) (indicated by the differential regulation of *Pltp, Apof, Lyz, Apoa* and *Agt* in estrus; *Smarcd3, Crabp2, Aldh1a1, Scand1, Rpl7a* and *Rarg* in diestrus) is altered in GCs of FL1 mice in both stages of estrus cycle.

Furthermore, xenobiotic metabolism (indicated by the differential expression of *Map3k14*, *Pik3c2g*, *Mgst2*, *Maf* and *Gsto1*) is predicted to be induced in GCs of FL1 mice in estrus. Also in diestrus, xenobiotic metabolism signaling (*Mgst2*, *Hs3st1*, *Maf*, *Aldh1a1*, *Scand1*) is predicted to be altered in GCs of FL1 mice compared to ctrl mice.

While some functional pathways are predicted to be differentially regulated in both stages of estrous cycle, the modifications of others are restricted to only on stage of estrous cycle. For instance, gluconeogenesis (indicated by the differential expression of *Gapdh, Me1* and *Me2*) and in the inhibition of matrix metalloproteases (indicated by the differential expression of *Timp1, Mmp11* and *Lrp1*) in GCs of FL1 mice are only altered in estrus.

Furthermore, insulin receptor signaling (indicated by the differential expression of *Ppp1r14a, Pik3c2g, Sgk1* and *Inpp5b*) is predicted to be activated in GCs of FL1 mice in estrus, but not in diestrus, while the oxytocin signaling pathway (indicated by the differential expression of *Ptgfr, Ppara, Pla2g12a, Pla2g4a* and *Cacna2d1*) and p38 MAPK signaling (indicated by the differential expression of *Pla2g12a, Pla2ga4* and *Mef2d*) are predicted to be altered only in GCs from diestrus of FL1 mice compared to ctrl mice.

### Differentially regulated biofunctions in FL1 mice versus ctrl mice

The functional annotation of differentially regulated genes in estrus and diestrus are provided in Table 4 and Table 5, respectively.

**Table 4:** Functional annotations of differentially expressed genes of FL1 mice in estrus. The biofunctions are calculated based on the IPA Ingenuity Knowledge Base. Official gene symbols are sorted by the fold change, starting with the lowest Log2FC

Functional annotation	*p*-value	z-score	Involved genes	
			Stronger expression	Weaker expression
Reproductive system development and function				
Fertility	3.66E-03	2.3	*F13a1, Clcf1, Kl, Pltp, Lhcgr, Timp1, Acsl4, Sfrp4, Mfge8*	*Inpp5b, Agt*
Litter size	1.74E-03	1.7	*Oxtr, Timp1, Acsl4, Efna5, Sfrp4, Setd3, Mfge8*	*Etnk2*
Lipid metabolism and molecular transport				
Synthesis of lipid	5.37E-07	1.0	*Map 3 k4, Elovl7, Pltp, Akr1c18, Rab27a, Lhcgr, Pik3c2g, Prlr, Star, Mgst, Fdx1, Me1, Lpin1, Idh1, Acsl4, Abcb1b, Sgms2, Tspo, Hsd3b1*	*Me2, Serinc5, Lta4h, Apoa4, Agt, Etnk2, Hpgd, St3gal5, Grem2, Neu3, Cyp1b1, Sgpp2, Dkkl*
Transport of lipid	1.79E-05	1.1	*Pcolce2, Pltp, Apof, Vnn1, Star, Lipg, Gm2a, Acsl4, Abcb1b, Nceh1, Tspo*	*Lrp1, Apoa4*
Transport of metal	7.19E-04	2.1	*Slc11a1, Cybrd1, Kcnab3, Prlr, Sgk1, Mt2, Ndrg2, Atp1b1, Nipal1, Mt1*	*Necitin1, Agt, Kcnj2, Prss30, H2-Q10*
Transport of molecule	1.23E-03	1.5	*Cym, Pcolce2, Ptpn22, Kl, Slc11a1, Pltp, Oxtr, Apof, Rab27a, Lhcgr, Cybrd1, Kcnab3, Prlr, Vnn1, Star, Lipg, Gm2a, Sgk1, Acsl4, Mt2, Abcb1b, Nipal1, Slc7a8, Nceh1, Sgms2, Mt1, Tspo, Ptp4a2*	*Lrp1, Upf1, Necitin1, Apoa4, Agt, Kdm8, Kcnj2, Prss30, H2-Q10*
Cell death and survival				
Apoptosis	2.07E-04	−1.8	*Clcf1, Ptpn2, Kl, Map 3 k14, Sema3f, Adamtsl4, Tub, Ifi203, Rab27a, Tnfrsf19, Lhcgr, Parvb, Lgals3, Rora, S100a4, Prlr, Vnn1, St3gal, Star, Sgk1, Eif4a1, Gapdh, Maf, Timp1, Ltbp1, Idh1, Dpp7, Acsl4, Mt2, Ethe1, Ndrg2, Sfrp4, Anxa5, Nek7, Nceh1, Sgms2, Nrp1, Mt1, Tspo, Ptp4a2, Mfge8, Pros1, Ubr4, Tcf4*	*Wdr5, Dynll1, Itsn1, Bcl11a, Ddit4, Lrp1*
Regeneration of cells	2.82E-04	0.7	*Clcf1, Sgk1, Mt2, Mt1, Tcf4*	*Agt, Neu3, Kcnj2*
Endocrine system development, function and disorder				
Steroid metabolism	6.89E-07	1.3	*Pltp, Akr1c18, Apof, Lhcgr, Rora, Prlr, Star, Fdx1, Acsl4, Tspo, Hsd3b1*	*Lrp1, Apoa4, Agt, Grem2, Cyp1b1*
Metabolism of progesterone	2.89E-06	1.3	*Akr1c18, Lhcgr, Prlr, Star, Acsl4, Tspo, Hsd3b1*	*Agt, Grem2*
Metabolism of hormone	5.12E-07	1.3	*Crym, Elovl7, Akr1c18, Lhcgr, Prlr, Star, Fdx1, Acsl4, Tspo, Hsd3b1*	*Agt, Grem2, Cyp1b1, Dkkl*
Reduction of hormone	3.65E-03	n.a.	*Akr1c18, Hsd3b1*	
Hypogonadism	2.82E-04	n.a.	*F13a1, Kl, Sema3f, Lhcgr, Prlr, St3gal1, Ltbp1*	*Serinc5*

**Table 5:** Functional annotations of differentially expressed genes of FL1 mice in diestrus. The biofunctions are calculated based on the IPA Ingenuity Knowledge Base. Official gene symbols are sorted by the fold change, starting with the lowest Log2FC

Functional annotation	*p*-value	z-score	Involved genes	
			Stronger expression	Weaker expression
Lipid metabolism and molecular transport				
Quantity of steriod	1.03E-03	0.9	*Star, Ptgfr, Ppara, Maf, Hsd11b2, Gpm6b, Bambi, Scarb1, Angptl4, Atp1a1*	*Epthx, St3gal5*
Synthesis of lipid	1.03E-06	0.6	*Inpp5d, Runx2, Star, Tnfsf10, Mgst2, Ppara, Pla2g12a, Rab27a, Hsd11b2, Pla2g4a, Slc6a6, Scarb1, Angptl4, Aldh1a1, Atp1a1, Ctnnb1*	*Me2, Ephx2, Hpgd, Neu3, Grem2, St3gal5, Il15*
Concentration of lipid	2.53E-06	2.6	*Inpp5d, Star, Tnfsf10, Ptgfr, Ppara, Smarcd3, Rgcc, Rab27a, Maf, Hsd11b2, Gpm6b, Bambi, Pla2g4a, Scarb1, Angptl4, Aldh1a1, Atp1a1, Ctnnb1*	*Ephx2, Cacna2d1, Hpgd, Neu3, St3gal2*
Synthesis of fatty acid	3.30E-04	1.8	*Tnfsf10, Mgst2, Pla2g4a, Angptl14, Aldh1a1, Ctnnb1,*	*Me2, Ephx2, Hpgd, Il15*
Lipolysis	4.77E-04	1.9	*Inpp5d, Tnfsf10, Ppara, Gm2a, Pla2g4a, Scarb1, Angptl4*	*Neu3*
Hydrolysis of lipid	1.78E-04	1.6	*Inpp5d, Tnfsf10, Gm2a, Pla2g4a, Scarb1, Angptl4*	*Neu3*
Cell death and survival				
Apoptosis	5.01E-04	0.4	*Cd53, Inpp5d, Atp6v0d2, Runx3, Dusp2, Runx2, Tcim, Tnfaip2, Ptprz1, Star, Tnfsf10, Ptgfr, Parvb, Ppara, Rab27a, Maf, Hsd11b2, Gpm6b, Rnase1, Mmd2, Pla2g4a, Sema7a, Crabp2, Ccng1, Slc6a6, Scarb1, Angptl4, Aldh1a1, Atp1a1, Ctnnb1*	*Triap1, Mef2d, Hspe1, Mrtfa, Rarg, Hpgd, Neu3, Rassf2, Gjc1, Il15, Dph7*

The transcriptome analysis indicates that a large number of genes associated with increased litter size such as the luteinizing hormone receptor (*Lhcgr*) and Klotho (*Kl*) are higher expressed in GCs of FL1 mice compared to ctrl mice in estrus (Table 4). Also several genes associated with increased litter size, including the oxytocin receptor (*Oxtr*) and SET domain containing 3 (*Setd3*) are higher expressed in GCs of FL1 mice in estrus (Table 4). The tissue inhibitor of metalloprotease 1 (*Timp1*), the acyl-CoA synthetase long-chain family member 4 (*Acsl4*), and the serum and glucocorticoid regulated kinase 1 (*Sgk1*) are higher expressed in GCs of FL1 mice in estrus. Both genes play a role in the increased fertility as well as in the increased litter size (Table 4). Furthermore, these and other genes (see Table 4) are involved in development, function and disorder of the endocrine system such as synthesis and metabolism of steroid hormones. Apoptosis has been predicted for the GCs of FL1 mice as significantly decreased by the factor − 1.8 in estrus. Moreover, the molecular transport, especially the transport of lipid, metal and hormones is increased in the GCs in FL1 mice in estrus.

Common biofunctions in both estrus and diestrus of both mouse lines are the regulation of the survival of cells, as well as the assembly and organization of cells. The lipid metabolism is also altered in estrus, as well as in diestrus in GCs of FL1 mice. Catenin beta-1 (*Ctnnb1*) is higher expressed in GCs of FL1 mice in diestrus and regulates different traits of lipid metabolism and the synthesis of fatty acids. Also the steroidogenic acute regulatory protein (*Star*) as well as the 11-beta-hydroxysteroid dehydrogenase type 2 (*Hsd11b2*) are higher expressed in GCs of FL1 mice and are relevant genes in the lipid as well as in the steroid metabolism.

### Hormonal analysis

In addition, several parameters associated with the ascertained pathways and functional annotations were analyzed on endocrine and/or molecular levels to obtain a global picture of how the physiological mechanisms are altered in FL1 mice. Since the results of the transcriptome analysis indicate that the alterations in GCs of FL1 mice are associated with increased reproductive performance predominantly in estrus, we mainly focused on this stage of estrus cycle.

**Fig. 2:**
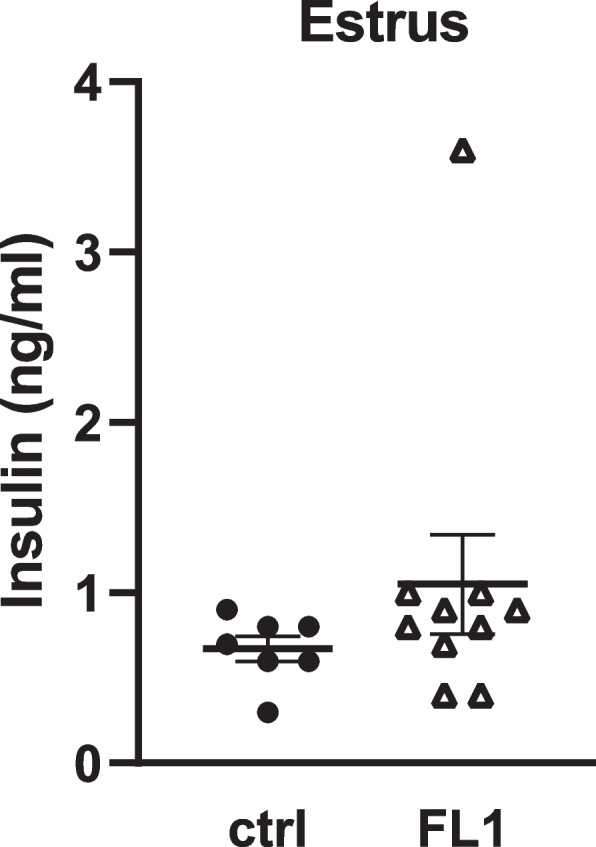
Serum concentration of insulin in ctrl mice and FL1 mice in estrus; Data are expressed as mean ± S.E.M (Mann-Whitney-U-Test)

Insulin receptor signaling is predicted to be activated in GCs of FL1 mice in estrus (Table 2). Plasma concentrations of insulin were measured in estrus. The results are shown in Fig. 2. We found no significant difference in the plasma concentrations of insulin between FL1 mice (1.05 ± 0.3 ng/ml) and ctrl mice (0.67 ± 0.07 ng/ml).

**Fig. 3:**
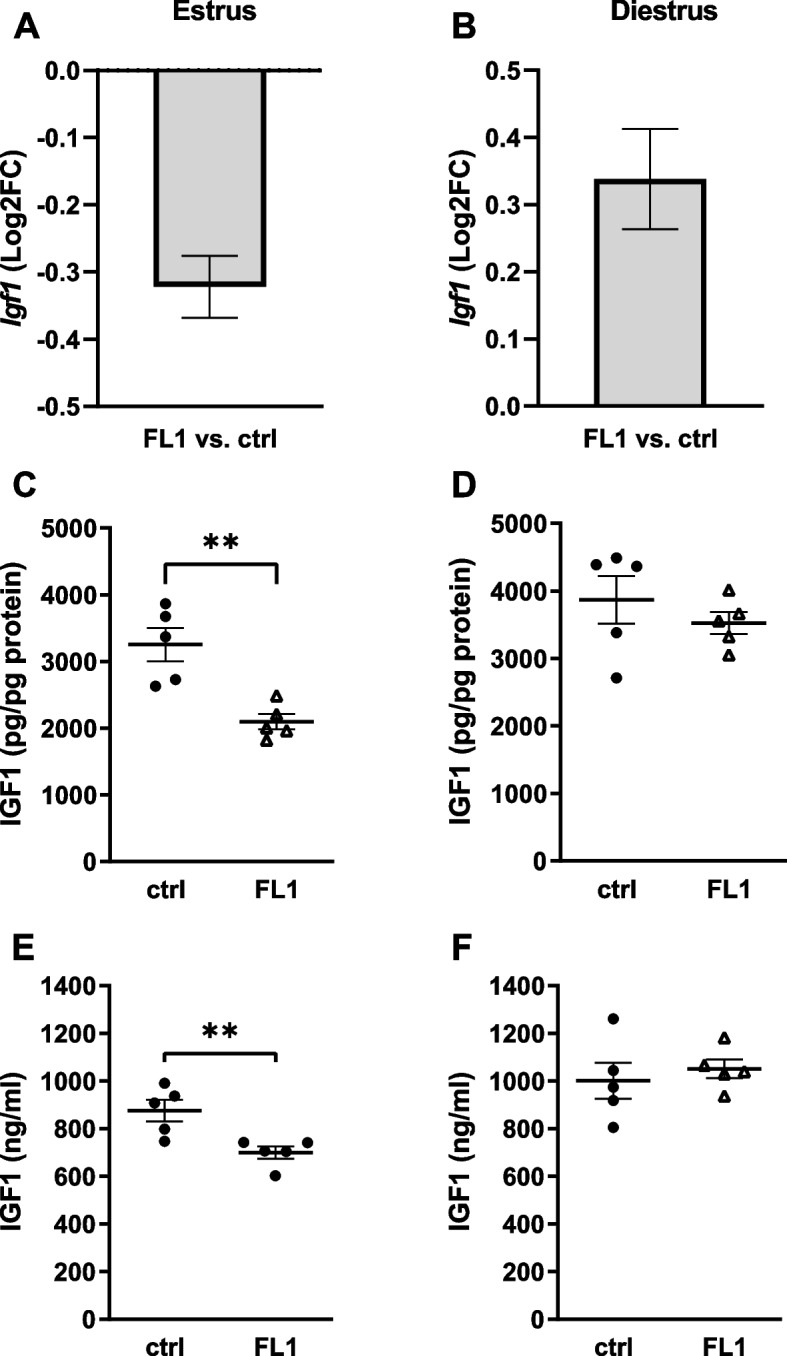
Levels of IGF1 in ctrl mice and FL1 mice in estrus and diestrus. mRNA expression of *IFG1* in the liver of ctrl mice and an FL1 mice in estrus (**A**) and diestrus (**B**); Data are expressed as Log2FC ± S.D., ** *p* < 0.01. IGF1 protein content (pg/pg total protein) in ctrl mice and FL1 mice in estrus (**C**) and diestrus (**D**); Data are expressed as mean ± S.E.M, ** *p* < 0.01 (Mann-Whitney-U-Test). Serum concentration of IGF1 (ng/ml) in ctrl mice and FL1 mice in estrus (**E**) and diestrus (**F**); Data are expressed as mean ± S.E.M, ** *p* < 0.01 (Mann-Whitney-U-Test)

We found no significant difference in *Igf1* gene expression in the liver between FL1 mice and ctrl mice in estrus (Fig. 3A) or in diestrus (Fig. 3B). However, the expression of *Igf1* tended to be decreased in the liver of FL1 mice in estrus (Fig. 3A). The protein concentration of IGF1 in the liver in estrus was significantly decreased in FL1 mice compared to ctrl mice (Fig. 3C). Also serum concentrations of IGF1 were significantly decreased in FL1 mice (699.0 ± 25.6 ng/ml) compared to ctrl mice (875.6 ± 45.1 ng/ml) in estrus. Our results revealed no significant difference in IGF1 protein concentrations in the liver or IGF1 plasma concentrations between FL1 mice and ctrl mice in diestrus (Fig. 3 D and E).

**Fig. 4:**
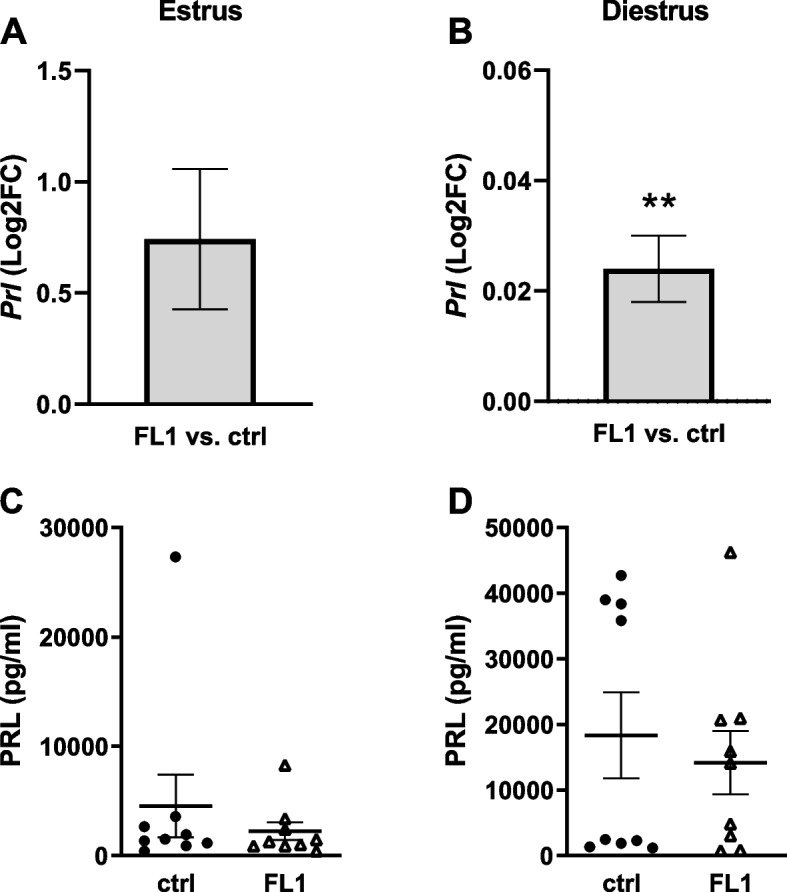
Levels of PRL in ctrl mice and FL1 mice in estrus and diestrus. mRNA expression of *Prl* in the pituitary of ctrl mice and an FL1 mice in estrus (**A**) and diestrus (**B**); Data are expressed as Log2FC ± S.D., ** *p* < 0.01. Serum concentration of PRL (pg/ml) in ctrl mice and FL1 mice in estrus (**C**) and diestrus (**D**). Data are expressed as mean ± S.E.M (Mann-Whitney-U-Test)

PRL levels were analyzed on transcriptional and plasma levels in estrus and diestrus. We found no significant difference in *Prl* expression in the pituitary between FL1 and ctrl mice in estrus (Fig. 4A). However, in diestrus *Prl* expression was slightly, but significantly increased in diestrus (Fig. 4B). PRL serum concentrations were not significantly different between ctrl mice and FL1 mice in estrus (Fig. 4C) and diestrus (Fig. 4D).

**Fig. 5:**
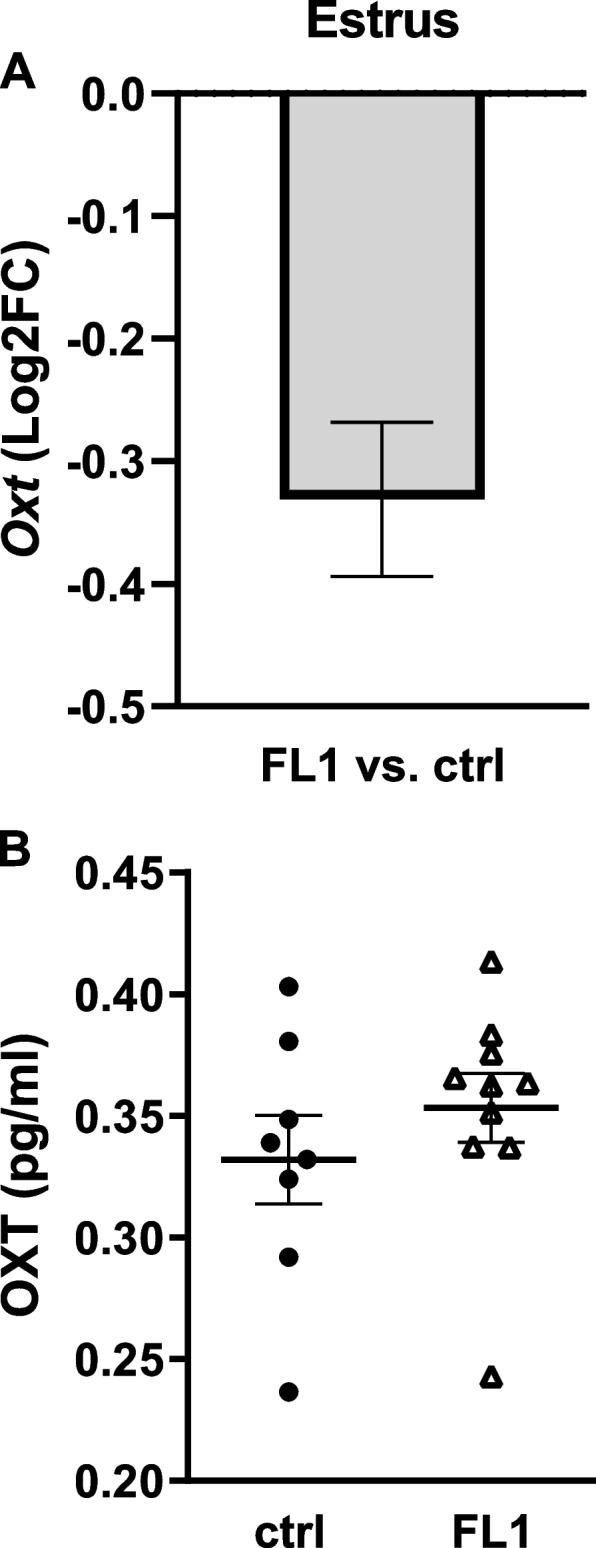
Levels of OXT in ctrl mice and FL1 mice in estrus. mRNA expression of *Oxt* in the hypothalamus of ctrl mice and an FL1 mice in estrus (**A**); Data are expressed as Log2FC ± S.D. Plasma concentration of OXT (pg/ml) in ctrl mice and FL1 mice in estrus (**B**). Data are expressed as mean ± S.E.M (Mann-Whitney-U-Test)

OXT was measured on transcriptional (Fig. 5A) level in die hypothalamus and plasma level (Fig. 5B) in estrus. Neither *Oxt* gene expression, nor plasma levels of OXT differed between FL1 mice and ctrl mice in estrus.

## Discussion

The Dummerstorf high fertility mouse line 1 is a worldwide unique animal model for increased reproductive performance. These mice did not only increase the ovulation rate, they are furthermore able to deliver high litter sizes over a long time period without health issues [22]. Although it is widely assumed that FSH is one of the most important key regulators in antral follicle development, FL1 mice exhibit FSH levels which are decreased by more than 50% compared to unselected ctrl mice [21]. Thus, we have to reconsider the role of FSH in folliculogenesis in connection with improved fertility. To gain insights into the mechanisms, leading to the increased reproductive performance of FL1 mice despite decreased FSH levels, we analyzed GCs isolated from antral follicles on molecular levels. Based on the results of the transcriptome analysis we measured endocrine parameters associated with follicular growth and GC differentiation to draw a global picture of how the phenotype of high fertility is achieved.

The transcriptome analysis indicated that 25% of all differentially expressed genes in GCs of FL1 mice compared to ctrl mice in estrus were associated with reduced apoptosis. Considering the fact that more than 99% of the antral follicles undergo atresia and do not enter ovulation [6, 7], decreasing GC death to improve follicular survival is an obvious opportunity to increase ovulation rates. Although it is widely believed that FSH is the most important key regulator in promoting follicular survival by inhibiting GC apoptosis is FSH [10–13], other factors are also able to regulate the survival of GCs. A variety of different parameters, including several genes, as well as other hormones and growth factors, are able to modulate different signaling pathways associated with apoptotic processes.

One of these genes that is associated with decreased apoptosis is *Timp1*. It is higher expressed in GCs of FL1 mice in estrus. By inhibiting the matrix metalloproteases and therefore remodeling the extracellular matrix, *Timp1* has several important functions in the control of cell proliferation, cell survival, ovulation, steroidogenesis, and in the regulation of the estrous cycle [23–26]. In addition, Nothnick et al. (2001) showed that a inhibition of *Timp1* is associated with a reduced reproductive lifespan of female mice [27]. Interestingly, it has been shown that the expression of *Timp1* is dependent on FSH. During follicular development, *Timp1* expression can be regulated directly by the PKA signaling pathway or indirectly through IGF1 [26]. Strikingly, FSH levels are significantly decreased in FL1 mice [21] and also IGF1 levels are significantly lower in FL1 mice in estrus. The transcriptome analysis revealed no evidence that the receptors of FSH or IGF1 are differentially regulated in FL1 mice. Thus, an upregulation of *Timp1* by FSH, IGF1 or an increased receptor expression of these two hormones is not likely. Thus, other activators of FSH target gene expression must be involved.

Above, *Timp1* also other important and well described (anti-apoptotic) FSH and IGF1 target genes are not inhibited despite decreased hormonal levels. Quite the opposite is the case.

*Lhcgr*, which is a very well described FSH target gene is higher expressed in the GCs of FL1 mice. It plays a crucial role during antral follicle formation and a great number of activating or inactivation mutations and also knockout models have been described in literature [28]. Since not only *Lhcgr* is higher expressed in GCs of FL1 mice, but also the transcript levels of *Lh* in the pituitary, as well as the LH serum concentrations are increased in FL1 mice compared to ctrl mice [21], our results indicate that increased LH levels and receptor signaling might play a crucial role in the improved follicular development of FL1 mice and especially have the potential to improve antral follicle formation. Interestingly, AKT is the preferred target of LH [29]. Thus, increased LH levels represent a possibility to induce FSH and target genes and intracellular signaling pathways and therefore might compensate the decreased FSH levels in FL1 mice.

Also *Sgk1,* which is significantly higher expressed in GCs of FL1 mice in estrus is an important FSH target gene, which has several effects on the proliferation and survival of cells [30]. Alliston et al. (1997) analyzed the role of *Sgk1* in the proliferation and differentiation of rat GCs and showed that *Sgk1* expression in GCs is regulated by FSH. The authors concluded that the expression of *Sgk1* in GCs is induced by FSH via activators of the PKA pathway [31]. However, like previously described, FSH is significantly decreased in FL1 mice compared to ctrl mice [21]. However, FSH is not the only hormone that has the ability to regulate *Sgk1* expression. Also insulin has been shown to activate *Sgk1* via PI3K signaling [32]. Although plasma levels of insulin do not differ significantly between FL1 and ctrl mice, the fact that several genes associated with increased insulin signaling are differentially expressed in GCs of FL1 mice suggests a role of insulin in the increased ovulation rate of FL1 mice. The two main intracellular Insulin receptor signaling pathways are the PI3K/AKT and the MAPK signaling pathway [2, 33], which can also be induced by FSH and LH.

The fact that numerous well described FSH target genes are higher expressed in FL1 mice and also important FSH induced signaling pathways are not inhibited despite decreased FSH levels in FL1 mice promotes the hypotheses that other activators of gene expression are involved in these processes. Thus, although FSH is undoubtedly important as part of the HPG axis and is clearly important for adequate follicular development, other signal transduction pathways must be present to compensate for low FSH and maintain superior fertility in FL1 females.

In addition to LH, also a role of PRL and OXT in this complex interaction of anti-apoptotic signaling pathways is conceivable. In view of the fact that PRL and OXT signals can be mediated by various pathways including the MAPK signaling pathway and the PI3K signaling pathway, it promotes the hypothesis that the increased expression of *Prlr* and *Oxtr* in GCs of FL1 mice in estrus might be involved in the alternative way of signaling by activating antiapoptotic pathways in the GCs.

While it has already been shown that PRL might act as a survival factor in GCs [34], the role of OXT in the folliculogenesis is controversially discussed in literature. Although several studies assume that OXT plays a crucial role in reproductive physiology, the role of OXT in folliculogenesis is not clear. Female mice lacking OXT are fertile and able to deliver pups [35]. Administration of OXT has no effects on the number of follicles and the ovulation rate [36, 37], but the abundance of *Oxtr* mRNA in GCs suggests a role of OXT in follicular development. OXT has been shown to stimulate the production of LH in the pituitary [38]. Conveniently, mRNA levels of LH are increased in FL1 mice in estrus [21]. OXT increases the volume of ovaries, corpora lutea as well as the endometrial thickness [36] and therefore might support ovulation and late follicular maturation. Chandrasekher et al. analyzed the effects of OXT on follicle cells in vitro. GCs treated with OXT increased the production of progesterone. Interestingly, this effect was only visible in a medium without FSH. The authors concluded, that either the stimulatory effects of OXT on progesterone production might not be expressed in the presence of the maximally stimulatory effects of FSH used in these experiments or that high levels of intracellular OXT, produced in the presence of FSH, masked the effects [39]. Therefore, interactions of altered progesterone, OXT and FSH levels might play a major role in the increased reproductive performance of FL1. However the mRNA content of *Oxt* in the hypothalamus and the OXT plasma concentrations revealed no significant difference between FL1 and ctrl mice. Thus, the actions of OXT appear to be restricted local to the ovary.

In addition to insulin, also other growth factors are able to control apoptosis of GCs and follicular development. IGF1 has long been recognized to have an indispensable role in female reproductive performance. *Igf1* as well as *Igf1r* knockout mice have reproductive defects and are infertile [40, 41]. Kadakia et al. (2001) demonstrated, that IGF1 is necessary for the proliferation of GCs [3]. In addition, IGF1 signaling is essential for the FSH induced stimulation of AKT in the GC [42] and also the fact that *Igf1* knockout mice phenocopy *Fsh* as well as *Fshr* knockout mice [1] demonstrates the great correlation between IGF1 and FSH signaling. Concurrently, the close connection and similarity of FSH and IGF1 signaling suggests that not only high FSH levels, but also high IGF1 levels might awake a “dark side” and therefore it is consistent that decreased FSH levels are correlated to decreased IGF1 levels in FL1 mice.

**Fig. 6:**
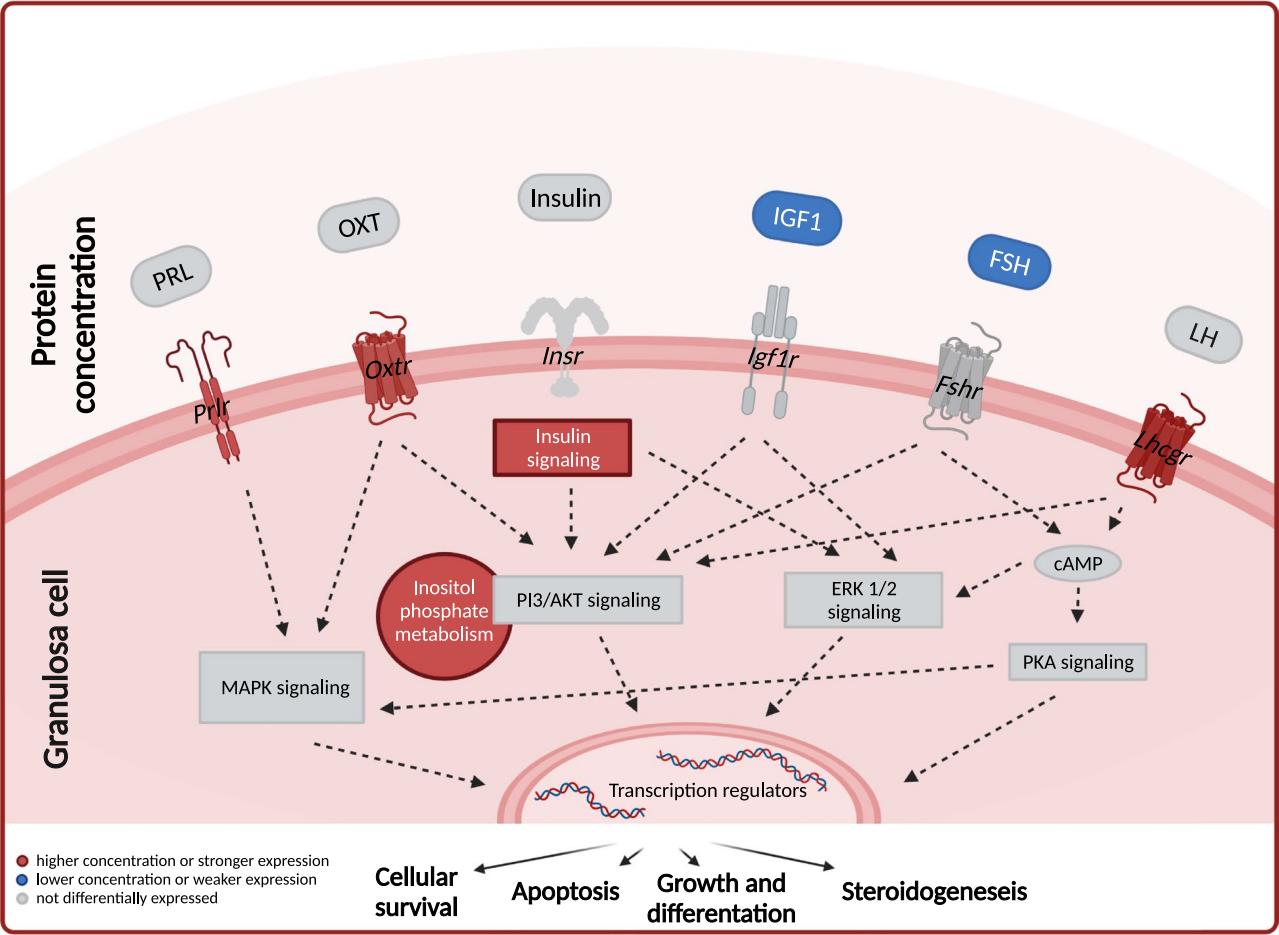
Signaling pathways associated with follicular development in GCs of FL1 mice. Although FSH levels in FL1 females are significantly decreased, the pathway analysis indicates that the most important intracellular FSH signaling pathways in the GCs are not inhibited. Thus, the regulation of important functions such as cellular survival, apoptosis, growth and differentiation and also steroidogenesis in the GCs of FL1 mice might be modulated by factors except for FSH. An overlap of growth factor and gonadotropin (receptor) signaling provides opportunities for cross-talk between insulin, IGF1, PRL, OXT, FSH and LH signaling. Furthermore, the induction of important anti-apoptotic signals by other factors has the potential to preserve FL1 mice from negative effects of high FSH and IGF1 levels. Data are partly extracted from Ludwig et al. 2022. Figure created with BioRender.com

Hence, the overlap of different signaling pathways not only provides the opportunity for (improved) cross-talk between gonadotropin and growth factor signaling pathways and in order to enhance intracellular signaling in the GCs and compensate the decreased FSH levels in FL1 mice, it furthermore has the potential to preserve FL1 mice from the negative effects of high FSH levels such as accelerated reproduction failure, premature infertility and ovarian failure [16, 43]. A graphical summary of how the signaling pathways might interact is given in Fig. 6.

As recently described, the litter size of FL1 mice is not only initially increased in primiparous females, they are able to deliver high litter sizes with progressive age and show no signs of health issues or decreased live expectancy despite this remarkable reproductive performance [17, 22]. Decreased IGF1 levels might not only have advantages in reproductive performance, but also in life expectancy and health. Although fertility and reproduction are not affected in female IGF1R+/− mice, they are characterized by an increased lifespan and are able to live 33% longer than the controls [44]. Also Kurosu et al. (2005) showed that inhibition of IGF1 signaling prolongs the life span of mice. In their experiments, the inhibition of IGF1 signaling was obtained by overexpressing of *Kl* [45]. Interestingly, *Kl* is higher expressed in GCs FL1 mice compared to ctrl mice. Therefore, it is not only consistent that decreased IGF1 levels in FL1 mice are related to decreased FSH levels and vice versa, furthermore the anti-aging hormone *Kl* might be involved in these signaling pathways.

The ovulation rate of FL1 mice might not only be increased due to decreased apoptosis of GCs, our results furthermore indicate that also other processes in the GCs of FL1 mice, such as proliferation, differentiation, signal transduction, molecular transport and metabolic control are also improved. These physiological events can be regulated by numerous different factors, but a very prominent one is the modulation of the intracellular Ca2+ concentration [46–48]. In the GCs, the inositol metabolism plays a central role in the mobilization of Ca2+ from the intracellular stores [48, 49]. Several genes associated with an increase of different traits of the Inositol metabolism are differentially regulated in GCs of FL1 mice in both estrus and diestrus. Furthermore, high inositol levels in the follicular fluid have been shown to be a marker for good oocyte quality and are involved in the maturation of follicles [50]. By modulating the Ca^2+^ signaling, retinoic acid has been shown to have stimulatory effects on the proliferation of GCs and therefore regulates the development of ovarian function [51]. Conveniently, several genes associated with the RAR activation are differentially expressed in GCs of FL1 mice in diestrus.

Our data indicate that the metabolism of hormones, especially the steroid and progesterone metabolism is induced in GCs of FL1 mice in estrus and also in diestrus we found several genes which are involved in the steroid metabolism. In addition there is not only evidence that the steroid metabolism is modified in FL1 on molecular levels, also P4 concentrations in serum are altered in FL1 mice. We recently showed that P4 serum concentrations are significantly decreased in FL1 mice [21]. Decreased P4 concentrations in FL1 might be caused by the decreased LXR/RXR activation in GCs of FL1 mice. Drouineaud et al. (2007) showed that treatment of GCs with an LXR agonist resulted in a dose-dependent reduction of the P4 secretion [52]. Thus LXR might be an important key player in the control of steroidogenesis. Also the results of Steffensen et al. (2006) showed that LRX affects ovarian function and is important in female reproductive performance. The female LXR knockouts conceived less frequently and had significantly fewer pups per litter [53].

Glucose is an important substrate for the generation of ATP. ATP is needed for metabolic as well as physiologic function of the cells and it nurtures follicular development. Armstron and Greep (1962) showed that LH increases the uptake of glucose [54]. Thus, increased LH levels and improved LH receptor signaling together with induced Insulin signaling might enhance the glucose uptake into the GCs and therefore ensure sufficient levels of substrate for the generation of ATP.

**Fig. 7:**
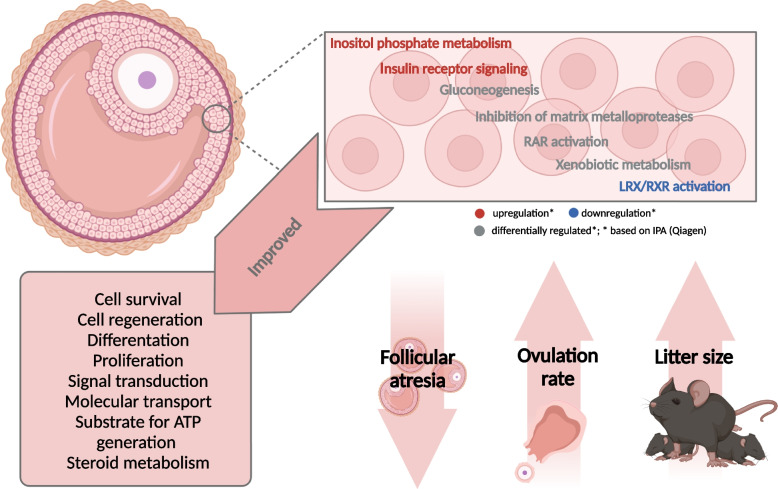
Improvement of intracellular mechanisms in GCs of FL1 mice. Gene expression data indicate alterations of different signaling pathways in the GCs of FL1 mice, which have the potential to improve important intracellular functions leading to decreased follicular atresia and therefore increase the litter size by enhancing the ovulation rate. Figure created with BioRender.com

Taken together our results indicate that (1) follicular atresia due to GC apoptosis might be decreased in FL1 mice due to the modulation of multiple genes and signaling pathways associated with the process of apoptosis, (2) the overlap of different signaling pathways provides the opportunity for cross-talk between gonadotropin and growth factor signaling pathways, which has not only the potential to compensate the decreased FSH levels in FL1 mice, but furthermore might preserve FL1 mice from the negative effects of high FSH levels, and (3) by improving the efficiency of intracellular signaling, glucose metabolism and signal transduction, FL1 mice have several advantages in reproductive performance compared to ctrl mice and therefore increased the ovulation rate. Thus, due to a complex combination of molecular and endocrine alterations, FL1 females respond with a higher ovulation rate as well as improved lifetime fecundity. A graphical summary of the alterations in GCs of FL1 mice is given in Fig. 7. The understanding of these mechanisms and their interplay might be of fundamental interest for the understanding of proper fertility in human and (farm) animals.

## Methods

### Animals and housing

The ctrl mouse line and the FL1 mouse line originally derived from the same genetic background. Both mouse lines are descendants of the same founder population, that was created by cross-breeding of four different inbreed and four outbreed mouse lines [55, 56]. The ctrl line did not undergo any selection process. To minimize the degree of kindship, a rotational mating scheme with 125–200 breeding pairs per generation has been used in the ctrl line. The FL1 line has been selected for the litter size and the total weight of the litters at birth of primiparous females (Dummerstorf fecundity index = 1.6x litter size + litter weight) for the first 162 generation. In the following generations the FL1 mice were selected for the Best Linear Unibased Prediction breeding value estimation, focusing only on the litter size. In total 60–100 breeding pairs per generation were used [20, 55].

All procedures were performed in accordance to national and international guidelines and approved by the institutional board (Animal Protection Board from the Research Institute for Farm Animal Biology) and the federal state of Mecklenburg Western-Pomerania, Germany (approval ID 7721.3–2-015/20).

Female ctrl and FL1 mice, bred at the Research Institute for Farm Animal Biology (FBN), Dummerstorf, Germany, were housed in groups of three animals per cage. A commercial breeding diet for rodents and water was provided ad libitum. Illumination of animal facilities was between 6.00 a.m. and 6.00 p.m. In addition, a male mouse was kept for acoustic, visual and olfactory stimulus in a separate cage.

### Determination of estrous cycle

The estrous cycle was determined like previously described [21]. A 10 μl drop of PBS (Roti®-CELL PBS, Carl Roth GmbH + Co. KG, Karlsruhe, Germany) was placed at the opening of the vaginal canal and was pipetted up and down three to four times. Then the recovered PBS drop, containing flushed cells of the vaginal mucosa, was placed on a glass slide and was evaluated under a light-microscope (Nikon ECLIPSE TE2000-S, 10x magnification) for the number, arrangement and density of cell types of the vaginal mucosa to determine the stage of estrous cycle. In this study we analysed samples, which were taken in estrus and diestrus. To keep the time point of sampling within estrus or diestrus as constant as possible, samples were taken when exclusively cornified epithelial cells (no other cells) appeared in vaginal smears in estrus, respectively only leucocytes in diestrus.

### Sample procedure

Animals at the age of 77 ± 5 days were euthanized by CO_2_ or nitrogen inhalation after the stage of the estrous cycle had been determined. After determination of death, the thorax was opened. The *Vena cava caudalis* was cut and blood was collected out of the thorax. To obtain serum, the blood samples were left for 2 h at room temperature, the resulting blood clot was removed and the supernatant was centrifuged (4 °C, 2000 g, 10 min). To obtain plasma, 8 μl EDTA (0.5 M, pH 8.0) were added to the blood samples. Pituitary and hypothalamus were extracted. Ovaries were removed and put into a petri dish with PBS (Roti®-CELL PBS, Carl Roth GmbH + Co. KG, Karlsruhe, Germany). 7–10 of the largest follicles were isolated. Each follicle was transferred into a PBS drop. The follicles were opened. It was taken care that the oocyte with the attached cumulus cells was not resorbed. Thus, only the mural granulosa cells were extracted and used for the transcriptome analysis. To separate the cells from residuals of follicular fluid and PBS they were centrifuged (4 °C, 21000 rpm, 10 min) and the supernatant was removed. All samples were immediately snap-frozen and stored at − 70 °C.

### Transcriptome analysis

GC samples of 10 animals per line (estrus *n* = 5; diestrus *n* = 5) were used for the transcriptome analysis. RNA was extracted with the RNeasy Plus Micro kit (Qiagen, Hilden, Germany). Libraries were prepared using the QuantSeq 3′ mRNA-Seq Fw. Library Prep kit (Lexogen) and sequenced to 10 M raw reads on an Illumina HiSeq 2500 machine, following the manufacturer’s protocols. Data were pre-processed and analyzed using the options recommended by the manufacturer. Trimmed sequences were aligned with STAR aligner 2.6.0a [57] against the GRCm38 (release 95) mouse genome retrieved from the Ensembl database via biomaRt [58] tool.

Raw sequence data, raw counts and metadata were deposited at the NCBI GEO repository, entry GSE209918. Differential analysis was performed with the DESeq2-package v1.14.1 [59] in R 4.0.0.

Validation experiments using qPCR to re-evaluate the results of the transcriptome analysis have recently been performed in our laboratory. The correlation between log2FC of the transcriptome sequencing and the qPCR analysis in estrus and in diestrus ranged between 0.93 and 0.98 [21].

The evaluation of the transcriptome analysis was performed using the Ingenuity Pathways Analysis software (IPA; Qiagen Bioinformatics software solutions) based on the Ingenuity Knowledge Base.

### qPCR

Liver samples of 10 animals (estrus *n* = 5; diestrus *n* = 5) were used for qPRC analysis of *Igf1*. Pituitary samples of 20 animals per line (estrus *n* = 10; diestrus *n* = 10) were used for qPCR analysis of *Prl*. Hypothalamus samples of 9 ctrl mice and 10 FL1 mice in estrus were used for qPCR analysis of *Oxt*.

Samples were pulverized in liquid nitrogen. RNA was extracted with the RNeasy Plus Micro Kit (Qiagen, Hilden, Germany) and reverse-transcribed using iScript cDNA Synthesis Kit (Biorad, München, Germany) according to the manufacturer’s protocol. Primers were designed using Primer-BLAST and purchased from TIB Molbiol (Berlin, Germany). Samples were analyzed by real-time PCR (iCycler, Biorad, München, Germany) in duplicates together with 4 μl primer mix, 5 μl iQ SYBR Green Supermix (Biorad, München, Germany) and 1 μl of cDNA reaction solution in a 96-well plate. The resulting copy numbers were corrected by a normalization factor based on the averaged expression of the reference genes *RPS18*, *36B4* and *B2m*. The sequences of the target and reference genes are shown in Table 6.

**Table 6:** Sequences of primers and reference genes

Gene	Primer sequence (5′-3′)	
	Sense	Antisense
*Prl*	AGC CCA GAA AGG GAC ACT CC	TGT TCC TTG TCT TCA GGT GTA GC
*Oxt*	GCCTGCTACATCCAGAACTGC	AGGCAGGTAGTTCTCCTCCTGG
*Igf1*	GTCGTCTTCACACCTCTTCTACCT	CAGTACATCTCCAGTCTCCTCAGA
*Rps18*	ACCATCATGCAGAACCCACGACAGT	CAGGTCCTCACGCAGCTTGTTGTCT
*36b4*	AAGCGCGTCCTGGCATTGTCT	CCGCAGGGGCAGCAGTGGT
*B2m*	TTCTGGTGCTTGTCTCACTGAC	GCAGTTCAGTATGTTCGGCTTC

### Hormonal analysis

Insulin was analyzed in duplicates in undiluted plasma samples using the Chrystal Chem High Performance Assay Ultra-Sensitive Mouse Insulin ELISA kit (Crystal Chem (Europe), Zaandam, Netherlands) following the manufacturer’s instructions on a FLUOstar Omega Microplate Reader. According to the manufacturer the sensitivity of the assay is 0.05 ng/ml. The intraassay variation below 10% is given by the manufacturer.

IGF1 serum levels as well as IGF1 protein content in the liver were measured in duplicates using the Mouse and Rat IGF1 Quantikine ELISA kit (Bio Techne GmbH, Nordenstadt, Germany). According to the manufacturer the sensitivity of the assay is 8.6 pg/ml. The intraassay variation is 4.1%.

For the measurement of PRL undiluted serum samples were assayed (repeated measurement) using an MPTMAG assay kit (Merck Millipore, Darmstadt, Germany) on a Luminex LX200 system according to the manufacturer’s instructions. According to the manufacturer the sensitivity of the assay is 34.1 pg/ml. The intraassay variation below 15% is given by the manufacturer.

For the measurement of OXT undiluted plasma samples were analyzed in duplicates using the Oxytocin ELISA Kit (OXT) (product number ABIN 365185, antibodies-online GmbH, Aachen, Germany) on a FLUOstar Omega Microplate Reader following the manufacturer’s instructions. According to the manufacturer the sensitivity of the assay is 0.6 pg/ml. The intraassay variation below 8% is given by the manufacturer.

### Statistical analysis

Relative gene expression and statistical analysis of the qPCR was calculated using the Relative expression software tool [60, 61]. Statistical analysis of the hormonal measurement was performed with the program GraphPad Prism 9.

## Data Availability

The datasets used and/or analyzed during the current study are available from the corresponding author on reasonable request.

## Abbreviations

AKT: Protein kinase B
Ctrl: Dummerstorf control mouse line
ERK1/2: Extra cellular-regulated kinases 1/2
FL1: Dummertorf high fertility mouse line 1
FSH: Follicle stimulating hormone
FSHR: Follicle stimulating hormone receptor
GC: Granulosa cell
HPG axis: Hypothalamus pituitary gonadal axis
IGF1: Insulin like growth factor 1
IGF1R: Insulin like growth factor 1 receptor
INSR: Insulin receptor
LH: Luteinizing hormone
LHCGR: Luteinizing hormone receptor
MAPK: Mitogen-activated protein kinase
OXT: Oxytocin
OXTR: Oxytocin receptor
PI3K: Phosphatidylinositol-4,5-bisphosphate 3 kinase
PKA: Protein kinase A
PRL: Prolactin
PRLR: Prolactin receptor

## References

[1] Pangas SA, Rajkovic A, Plant TM, Zeleznik AJ (2015). Chapter 21 - follicular development: mouse, sheep, and human models. Knobil and Neill's physiology of reproduction.

[2] Dupont J, Scaramuzzi RJ (2016). Insulin signalling and glucose transport in the ovary and ovarian function during the ovarian cycle. Biochem J.

[3] Kadakia R, Arraztoa JA, Bondy C, Zhou J (2001). Granulosa cell proliferation is impaired in the Igf1 null ovary. Growth Hormon IGF Res.

[4] Bachelot A, Monget P, Imbert-Bollore P, Coshigano K, Kopchick JJ, Kelly PA (2002). Growth hormone is required for ovarian follicular growth. Endocrinology.

[5] Bjersing L (1982). Maturation, morphology, and endocrine function of the ovarian follicle. Adv Exp Med Biol.

[6] Barnett KR, Schilling C, Greenfeld CR, Tomic D, Flaws JA (2006). Ovarian follicle development and transgenic mouse models. Hum Reprod Update.

[7] Matsuda F, Inoue N, Manabe N, Ohkura S (2012). Follicular growth and atresia in mammalian ovaries: regulation by survival and death of granulosa cells. J Reprod Dev.

[8] Matthews CH, Borgato S, Beck-Peccoz P, Adams M, Tone Y, Gambino G (1993). Primary amenorrhoea and infertility due to a mutation in the beta-subunit of follicle-stimulating hormone. Nat Genet.

[9] Casarini L, Crépieux P (2019). Molecular mechanisms of action of FSH. Front Endocrinol (Lausanne).

[10] Chu YL, Xu YR, Yang WX, Sun Y (2018). The role of FSH and TGF-β superfamily in follicle atresia. Aging (Albany NY).

[11] Zhou XL, Teng Y, Cao R, Fu H, Xiong K, Sun WX (2013). Rescue from dominant follicle atresia by follicle-stimulating hormone in mice. Genet Mol Res.

[12] Chun SY, Eisenhauer KM, Minami S, Billig H, Perlas E, Hsueh AJ (1996). Hormonal regulation of apoptosis in early antral follicles: follicle-stimulating hormone as a major survival factor. Endocrinology.

[13] Shen M, Liu Z, Li B, Teng Y, Zhang J, Tang Y (2014). Involvement of FoxO1 in the effects of follicle-stimulating hormone on inhibition of apoptosis in mouse granulosa cells. Cell Death Dis.

[14] Dierich A, Sairam MR, Monaco L, Fimia GM, Gansmuller A, LeMeur M (1998). Impairing follicle-stimulating hormone (FSH) signaling in vivo: targeted disruption of the FSH receptor leads to aberrant gametogenesis and hormonal imbalance. Proc Natl Acad Sci U S A.

[15] Peltoketo H, Strauss L, Karjalainen R, Zhang M, Stamp GW, Segaloff DL (2010). Female mice expressing constitutively active mutants of FSH receptor present with a phenotype of premature follicle depletion and estrogen excess. Endocrinology.

[16] McTavish KJ, Jimenez M, Walters KA, Spaliviero J, Groome NP, Themmen AP (2007). Rising follicle-stimulating hormone levels with age accelerate female reproductive failure. Endocrinology.

[17] Langhammer M, Michaelis M, Hoeflich A, Sobczak A, Schoen J, Weitzel JM (2014). High-fertility phenotypes: two outbred mouse models exhibit substantially different molecular and physiological strategies warranting improved fertility. Reproduction.

[18] Langhammer M, Michaelis M, Hartmann MF, Wudy SA, Sobczak A, Nurnberg G (2017). Reproductive performance primarily depends on the female genotype in a two-factorial breeding experiment using high-fertility mouse lines. Reproduction (Cambridge, England).

[19] Palma-Vera SE, Reyer H, Langhammer M, Reinsch N, Derezanin L, Fickel J (2022). Genomic characterization of the world’s longest selection experiment in mouse reveals the complexity of polygenic traits. BMC Biol.

[20] Spitschak M, Langhammer M, Schneider F, Renne U, Vanselow J (2007). Two high-fertility mouse lines show differences in component fertility traits after long-term selection. Reprod Fertil Dev.

[21] Ludwig CLM, Bohleber S, Rebl A, Wirth EK, Venuto MT, Langhammer M (2022). Endocrine and molecular factors of increased female reproductive performance in the Dummerstorf high-fertility mouse line FL1. J Mol Endocrinol.

[22] Langhammer M, Wytrwat E, Michaelis M, Schoen J, Tuchscherer A, Reinsch N, et al. Two mouse lines selected for large litter size display different lifetime fecundities. Reproduction (Cambridge, England). 2021. 10.1530/REP-20-0563 PMC8183634 33878028

[23] Hayakawa T, Yamashita K, Tanzawa K, Uchijima E, Iwata K (1992). Growth-promoting activity of tissue inhibitor of metalloproteinases-1 (TIMP-1) for a wide range of cells. A possible new growth factor in serum. FEBS Lett.

[24] Nothnick WB (2000). Disruption of the tissue inhibitor of Metalloproteinase-1 gene results in altered reproductive Cyclicity and uterine morphology in reproductive-age female Mice1. Biol Reprod.

[25] Gomez DE, Alonso DF, Yoshiji H, Thorgeirsson UP (1997). Tissue inhibitors of metalloproteinases: structure, regulation and biological functions. Eur J Cell Biol.

[26] Goldman S, Shalev E (2004). MMPS and TIMPS in ovarian physiology and pathophysiology. Front Biosci.

[27] Nothnick WB (2001). Reduction in reproductive lifespan of tissue inhibitor of metalloproteinase 1 (TIMP-1)-deficient female mice. Reproduction (Cambridge, England).

[28] Hunzicker-Dunn M, Mayo K, Neill JD (2006). CHAPTER 14 - gonadotropin signaling in the ovary. Knobil and Neill's physiology of reproduction.

[29] Casarini L, Lispi M, Longobardi S, Milosa F, La Marca A, Tagliasacchi D (2012). LH and hCG action on the same receptor results in quantitatively and qualitatively different intracellular signalling. PLoS One.

[30] Amato R, D'Antona L, Porciatti G, Agosti V, Menniti M, Rinaldo C (2009). Sgk1 activates MDM2-dependent p53 degradation and affects cell proliferation, survival, and differentiation. J Mol Med (Berl).

[31] Alliston TN, Maiyar AC, Buse P, Firestone GL, Richards JS (1997). Follicle stimulating hormone-regulated expression of serum/glucocorticoid-inducible kinase in rat ovarian granulosa cells: a functional role for the Sp1 family in promoter activity. Mol Endocrinol.

[32] Lang F, Shumilina E (2013). Regulation of ion channels by the serum- and glucocorticoid-inducible kinase SGK1. FASEB J.

[33] Diamanti-Kandarakis E, Papavassiliou AG (2006). Molecular mechanisms of insulin resistance in polycystic ovary syndrome. Trends Mol Med.

[34] Perks CM, Newcomb PV, Grohmann M, Wright RJ, Mason HD, Holly JM (2003). Prolactin acts as a potent survival factor against C2-ceramide-induced apoptosis in human granulosa cells. Hum Reprod.

[35] Lee H-J, Caldwell HK, Macbeth AH, Tolu SG, Young WS (2008). A conditional knockout mouse line of the oxytocin receptor. Endocrinology.

[36] Roshangar L, Soleimani Rad J, Nikpoo P, Sayyah MM (2009). Effect of oxytocin injection on folliculogenesis, ovulation and endometrial growth in mice. Int J Reprod BioMed.

[37] Robinson G, Evans JJ (1991). Oxytocin influences preovulatory follicular development and advances ovulation in rats. Acta Endocrinol.

[38] Robinson G, Evans JJ, Catt KJ (1992). Oxytocin stimulates LH production by the anterior pituitary gland of the rat. J Endocrinol.

[39] Chandrasekher YA, Fortune JE (1990). Effects of oxytocin on steroidogenesis by bovine theca and granulosa cells. Endocrinology.

[40] Baker J, Hardy MP, Zhou J, Bondy C, Lupu F, Bellvé AR (1996). Effects of an Igf1 gene null mutation on mouse reproduction. Mol Endocrinol.

[41] Baumgarten SC, Armouti M, Ko C, Stocco C (2017). IGF1R expression in ovarian granulosa cells is essential for steroidogenesis, follicle survival, and fertility in female mice. Endocrinology.

[42] Zhou P, Baumgarten SC, Wu Y, Bennett J, Winston N, Hirshfeld-Cytron J (2013). IGF-I signaling is essential for FSH stimulation of AKT and steroidogenic genes in granulosa cells. Mol Endocrinol.

[43] Nie X, Dai Y, Zheng Y, Bao D, Chen Q, Yin Y (2018). Establishment of a mouse model of premature ovarian failure using consecutive superovulation. Cell Physiol Biochem.

[44] Holzenberger M, Dupont J, Ducos B, Leneuve P, Géloën A, Even PC (2003). IGF-1 receptor regulates lifespan and resistance to oxidative stress in mice. Nature.

[45] Kurosu H, Yamamoto M, Clark JD, Pastor JV, Nandi A, Gurnani P (2005). Suppression of aging in mice by the hormone klotho. Science (New York, NY).

[46] Flores JA, Aguirre C, Sharma OP, Veldhuis JD (1998). Luteinizing hormone (LH) stimulates both intracellular calcium ion ([Ca2+]i) mobilization and transmembrane cation influx in single ovarian (granulosa) cells: recruitment as a cellular mechanism of LH-[Ca2+]i dose response. Endocrinology.

[47] Park DW, Cho T, Kim MR, Kim YA, Min CK, Hwang KJ (2003). ATP-induced apoptosis of human granulosa luteal cells cultured in vitro. Fertil Steril.

[48] Díaz-Muñoz M, de la Rosa SP, Juárez-Espinosa AB, Arellano RO, Morales-Tlalpan V (2008). Granulosa cells express three inositol 1,4,5-trisphosphate receptor isoforms: cytoplasmic and nuclear Ca2+ mobilization. Reprod Biol Endocrinol.

[49] Berridge MJ (2009). Inositol trisphosphate and calcium signalling mechanisms. Biochimica et Biophysica Acta (BBA) - molecular. Cell Res.

[50] Chiu TTY, Rogers MS, Law ELK, Briton-Jones CM, Cheung LP, Haines CJ (2002). Follicular fluid and serum concentrations of myo-inositol in patients undergoing IVF: relationship with oocyte quality. Hum Reprod.

[51] Demczuk M, Huang H, White C, Kipp JL (2016). Retinoic acid regulates calcium signaling to promote mouse ovarian granulosa cell proliferation. Biol Reprod.

[52] Drouineaud V, Sagot P, Garrido C, Logette E, Deckert V, Gambert P (2007). Inhibition of progesterone production in human luteinized granulosa cells treated with LXR agonists. Mol Hum Reprod.

[53] Steffensen KR, Robertson K, Gustafsson JA, Andersen CY (2006). Reduced fertility and inability of oocytes to resume meiosis in mice deficient of the Lxr genes. Mol Cell Endocrinol.

[54] Armstrong DT, Greep RO (1962). Effect of gonadotrophic hormones on glucose metabolism by luteinized rat ovaries. Endocrinology.

[55] Schüler L (1985). Selection for fertility in mice — the selection plateau and how to overcome it. Theor Appl Genet.

[56] Dietl G, Langhammer M, Renne U (2004). Model simulations for genetic random drift in the outbred strain Fzt:DU. Arch Anim Breed.

[57] Dobin A, Davis CA, Schlesinger F, Drenkow J, Zaleski C, Jha S (2013). STAR: ultrafast universal RNA-seq aligner. Bioinformatics.

[58] Smedley D, Haider S, Ballester B, Holland R, London D, Thorisson G, et al. BioMart--biological queries made easy. BMC Genomics. 2009;10:22. 10.1186/1471-2164-10-22 PMC2649164 19144180

[59] Love MI, Huber W, Anders S (2014). Moderated estimation of fold change and dispersion for RNA-seq data with DESeq2. Genome Biol.

[60] Pfaffl MW (2001). A new mathematical model for relative quantification in real-time RT-PCR. Nucleic Acids Res.

[61] Pfaffl MW, Horgan GW, Dempfle L (2002). Relative expression software tool (REST) for group-wise comparison and statistical analysis of relative expression results in real-time PCR. Nucleic Acids Res.

